# Atypical uterine leiomyoma: a case report and review of the literature

**DOI:** 10.1186/s13256-016-0800-3

**Published:** 2016-01-22

**Authors:** Suzana Manxhuka-Kerliu, Irma Kerliu-Saliu, Vjollca Sahatciu-Meka, Lloreta Kerliu, Labinot Shahini

**Affiliations:** 1 Faculty of Medicine, Institute of Pathology, University of Prishtina, Mother Theresa Street NN, 10 000 Prishtina, Kosovo; 2 Massachusetts College of Pharmacy and Health Sciences (MCPHS), 179 Longwood Avenue, Boston, MA 02115 USA; 3 Faculty of Medicine, University of Prishtina, Mother Theresa Street NN, 10 000 Prishtina, Kosovo

**Keywords:** Atypical uterine leiomyoma, Histology, Immunohistochemistry, Prognostic markers

## Abstract

**Background:**

Atypical uterine leiomyomas show benign behavior. However, the distinction between leiomyomas and leiomyosarcomas may at times be problematic. We report a rare case of atypical uterine leiomyoma. We try to investigate potential immunohistochemical parameters that could be essential to distinguish cases of malignant smooth muscle tumors and those of uncertain or borderline histology.

**Case presentation:**

A 56-year-old white ethnic Albanian woman from Kosovo presented with uterine bleeding because of uterine multiple leiomyomas. A hysterectomy with unilateral adnexectomy was performed. Her hysterectomy specimen contained multiple leiomyomas in submucosal, intramural and subserosal locations. The leiomyomas were well demarcated, firm and white with a whorled cut surface and one had foci of hemorrhage. Histology of most of the leiomyomas showed a whorled (fascicular) pattern of smooth muscle bundles separated by well-vascularized connective tissue. Smooth muscle cells were elongated with eosinophilic or occasional fibrillar cytoplasm and distinct cell membranes. Some of them developed areas of degeneration including hyaline change, with less than five mitotic figures per ten high power fields in most mitotically active areas, and no significant atypia. One leiomyoma was characterized by moderately to severely pleomorphic atypical tumor cells with low mitotic counts and no coagulative tumor cell necrosis. Immunohistochemistry showed strong immunoreactivity for vimentin, smooth muscle actin and desmin, while cyclin-dependent kinase inhibitor 2A (p16), and B-cell lymphoma 2 (bcl-2) showed focal immunoreactivity, estrogen and progesterone were positive, Ki-67 expressed a low proliferation index, whereas p21 and tumor suppressor gene p53 were negative.

**Conclusions:**

The combination of evaluation of conventional morphologic criteria with cyclin-dependent kinase inhibitor 2A (p16), p21, progesterone, B-cell lymphoma 2, tumor suppressor gene p53 and Ki-67 expression may be of great value in the assessment of uterine smooth muscle tumors of uncertain or borderline histology.

## Background

Atypical leiomyomas (ALMs) are characterized by moderately to severely pleomorphic atypical tumor cells with low mitotic counts and no coagulative tumor cell necrosis. Despite the worrisome histologic features, most tumors have shown benign behavior. However, most studied patients had total hysterectomies, and very few patients who had myomectomy alone have had long-term follow-up [1].

Individual features, such as hypercellularity, necrosis, nuclear atypia, mitotic figures, and intravascular growth, are ominous but must be interpreted with caution because variants of benign leiomyomas (LMs) may contain such changes [2].

ALM when unassociated with either coagulative tumor cell necrosis or a mitotic index in excess of 10 mitotic figures per 10 high power fields (HPFs) and cytological atypia, even when severe, is an unreliable criterion for identifying clinically malignant uterine smooth muscle tumors. These atypical cells have enlarged hyperchromatic nuclei with prominent chromatin clumping (often smudged). Large cytoplasmic pseudonuclear inclusions often are present. The atypical cells may be distributed throughout the LM (diffuse) or they may be present focally (possibly, multifocally). When the atypia is at most multifocal and the neoplasm has been completely sampled, such tumors are designated “ALM with minimal, if any, recurrence potential.” Such lesions have behaved benignly except for a single reported case [3].

There are studies suggesting that uterine tumors classified as smooth muscle tumors of uncertain malignant potential (STUMPs) using criteria proposed by Stanford investigators are usually clinically benign but should be considered tumors of low malignant potential because they can occasionally recur, in some cases, years after hysterectomy. Of note, the two recurrent tumors were the only ones that were strongly immunoreactive for cyclin-dependent kinase inhibitor 2A, multiple tumor suppressor 1 (p16) and p53, supporting earlier observations that these markers may be helpful in the prediction of the behavior of STUMPs. Patients diagnosed with STUMPs should receive long-term surveillance [4].

The p16 protein has been identified as a tumor suppressor protein, which binds specifically to the cyclin-dependent kinase 4 (CDK4), inhibiting the catalytic activity of the CDK4-cyclin D complex, and thereby acts as a negative cell cycle regulator.

It has been shown that p16 might play an important role in sarcomagenesis. Furthermore, p16 might be a useful immunohistochemical marker, which could help to distinguish cases of smooth muscle tumors in which histologic features are ambiguous or borderline, but the use of p16 in a diagnostic setting should await further clinical studies and clarification of the mechanisms [5].

In uterine leiomyosarcomas (LMSs) p16 is overexpressed compared with LMs, benign LM variants and STUMPs. In combination with p53 and Mib1 (a monoclonal antibody), p16 may be of value as an adjunct to morphological examination in the assessment of problematic uterine smooth muscle tumors, although further large-scale studies with follow-up are necessary to confirm this [6].

In cases in which the type of necrosis is uncertain (coagulative tumor cell versus hyalinized), the addition of p16 may aid in discerning a subset of STUMP that should be classified as LMS [7].

## Case presentation

A 56-year-old white ethnic Albanian woman from Kosovo presented with uterine bleeding because of uterine multiple LMs. A hysterectomy with unilateral adnexectomy was performed. Histological diagnosis was multiple uterine LMs and an atypical uterine LM.

### Macroscopy

Her hysterectomy specimen contained multiple LMs in submucosal, intramural and subserosal locations. The LMs were well demarcated, firm and white with a whorled cut surface and one had foci of hemorrhage.

### Histology and immunohistochemistry

**Fig. 1:**
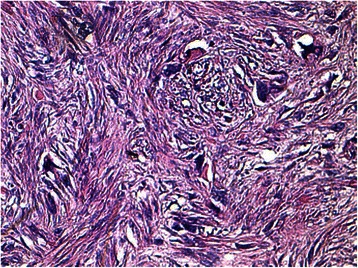
Atypical cells, hematoxylin and eosin stain 10×

**Fig. 2:**
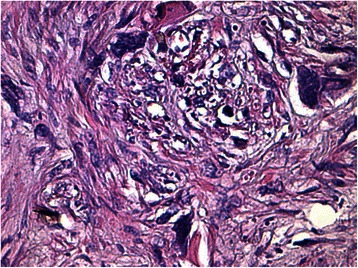
Atypical cells between fascicles of smooth muscle cells, hematoxylin and eosin stain 20×

**Fig. 3:**
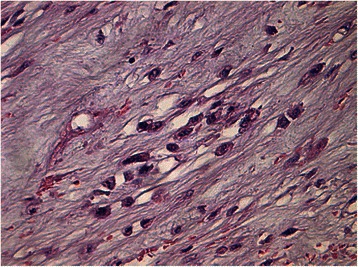
Atypical cells, hematoxylin and eosin stain 20×

**Fig. 4:**
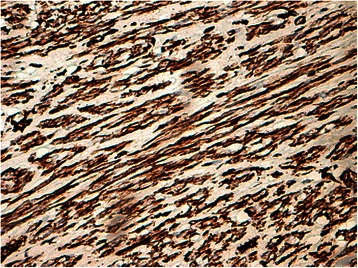
Vimentin^+^, 20×

**Fig. 5:**
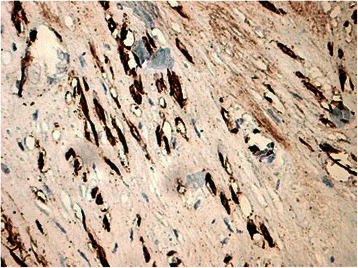
Smooth muscle actin^+^, 20×

**Fig. 6:**
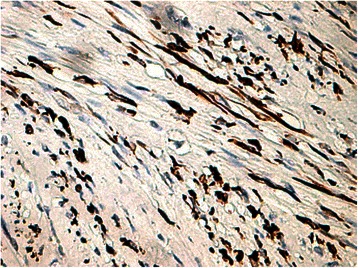
Desmin^+^, 20×

**Fig. 7:**
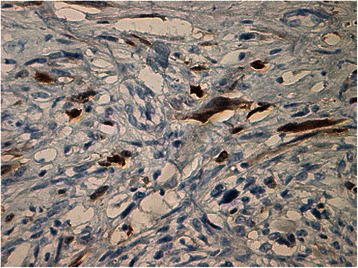
Cyclin-dependent kinase inhibitor 2A, focally positive, 20×

**Fig. 8:**
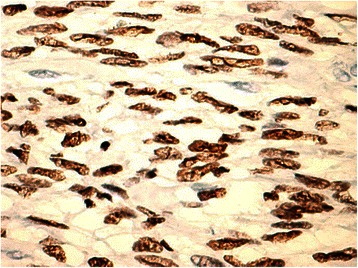
Estrogen^+^, 40×

**Fig. 9:**
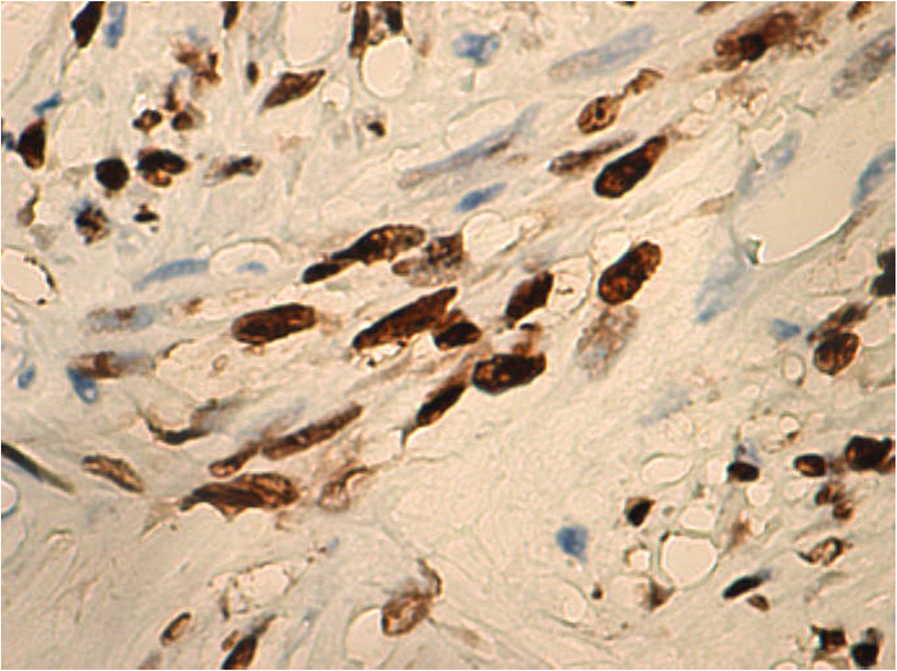
Progesterone^+^, 40×

**Fig. 10:**
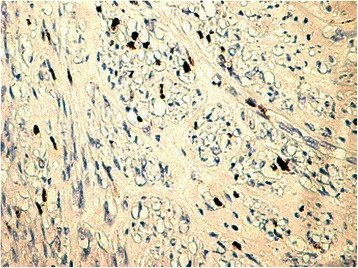
Ki-67 low proliferation index (10 %), 20×

Histology of most of the LMs showed a whorled (fascicular) pattern of smooth muscle bundles separated by well-vascularized connective tissue. Smooth muscle cells were elongated with eosinophilic or occasional fibrillar cytoplasm and distinct cell membranes. Some of them developed areas of degeneration including hyaline change, with less than five mitotic figures per ten HPFs in most mitotically active areas, and no significant atypia (Figs. 1, 2 and 3). One leiomyoma was characterized by moderately to severely pleomorphic atypical tumor cells with low mitotic counts and no coagulative tumor cell necrosis. Immunohistochemistry showed strong immunoreactivity for vimentin (Fig. 4), smooth muscle actin (SMA) (Fig. 5) and desmin (Fig. 6), while p16 (Cyclin-dependent kinase inhibitor 2A) showed focal immunoreactivity (Fig. 7), estrogen (ER) and progesterone (PR) were positive (Figs. 8 and 9), Ki-67 (a monoclonal antibody) expressed a low proliferation index (Fig. 10), whereas p21 and p53 were negative.

The final diagnosis of our case was ALM.

## Discussion

Among cases in which adjacent non-neoplastic tissue was well visualized, all were found to have pushing margins. The average tumor size was 6.8 cm. The patients’ average age was 42.5 years. In all cases, the initial diagnostic procedure was hysterectomy or myomectomy. ALM has a low rate of extrauterine intra-abdominal recurrence (<2 %) with a negligible risk for distant metastasis. Patients may be treated by myomectomy alone with successful pregnancy, but should be monitored for local intrauterine residual/recurrent disease [8].

In our case report, the tumor size was 5 cm, the patient’s age was 56 years and the diagnostic procedure was hysterectomy. We suggested follow-up of the patient in order to detect eventual recurrent tumor.

Cell cycle regulatory protein expression by immunohistochemical assay may have diagnostic utility in the distinction of uterine LMS from LM variants. Protein expression of p16, p21, p27 and p53 was evaluated by immunohistochemistry on 44 ALMs, 16 LMSs and eight cellular LMs. The ALM with extrauterine recurrence was diffusely positive for p21, but showed only weak focal (<33 %) staining for all other cell cycle markers [9].

We have evaluated p16 and p21 expressions in uterine smooth muscle tumors in order to determine whether p16 and p21 have a potential value in the differential diagnosis of problematic cases [10].

Likewise, our case showed focal staining for p16 and no staining for p21. In this way, these two cell cycle regulatory protein expression antibodies represented again their potential value in the distinction of ALM from LMS.

Bcl-2 protein is an apoptosis-inhibiting gene product that prevents the normal course of apoptotic cell death in a variety of cells. In addition, bcl-2 can promote cell replication by reducing the requirement for growth factors. This protein seems, therefore, to play an important role in the growth of tumors. The different expression of bcl-2 in uterine LMs, STUMPs and LMSs has been investigated.

Bcl-2 was expressed more frequently and more strongly in LMs compared with LMSs and STUMPs. The stronger bcl-2 expression in benign LMs and the better clinical outcome of bcl-2-positive LMS indicate that this protein seems to act as a good prognostic factor [11].

Similarly, bcl-2 in our case showed positive staining of atypical cells, supporting the diagnosis of ALM.

There are studies that have observed significant differences of steroid receptor expression between uterine LM, STUMP and LMS. The PR receptor may be an especially useful marker to distinguish cases of malignant smooth muscle tumors in which histological features are ambiguous or borderline [12].

Correspondingly, the results of our case report showed intense staining for PR and ER receptors that represented another proof of the benign nature of the lesion, contrary to LMS that has been shown to have weak or no expression of steroid receptors.

Ki-67 antigen expression may be a useful immunohistochemical parameter to distinguish between cases of malignant smooth muscle tumors and those of uncertain or borderline histology [13].

The Ki-67 proliferation index was very low (<10 %) in our case, which represented a reliable standard to differentiate the uncertain or borderline nature of the lesion from the LMS.

Significant differences were observed between LM and STUMP expression for Ki-67. We considered that more diagnostic criteria and parameters for diagnosis in doubtful cases among the three entities should be established. Immunoassaying for Ki-67, p53 and PR are such parameters. The panel of their expression in specific case eases diagnosis [14].

All LMs as well as ALMs and STUMPs were stained intensely for PR. Conversely, LMS was strongly stained with p53, while the three non-sarcomatous groups (STUMP, ALM and LM) were either entirely negative or weakly stained for p53. Combined high PR and low p53 expression was seen in all examined cases of the non-sarcomatous group including the STUMP cases and none of the LMS cases. Therefore, it represents a “benign” profile with 100 % specificity in diagnosis of a non-sarcomatous tumor [15].

Combined high PR and negative p53 expression has been shown in our case as well, helping us to exclude the sarcomatous nature of the lesion.

We did not observe p53 immunoreactivity in any of 18 (0 %) LMs, but we did observe it in one of six (17 %) STUMPs, and 16 of 34 (47 %) LMSs. Reactivity was not observed in the surrounding non-neoplastic uterine smooth muscle. Strong p53 overexpression in the LMSs was significantly associated with high-grade morphology (*P*=.013) and a high stage at the time of presentation (*P*=.021) [16].

The absence of p53 immunoreactivity in our case defined the benign profile of the tumor with high specificity.

## Conclusions

The distinction between ALMs, STUMPs and LMSs may at times be problematic. Therefore, the combination of evaluation of conventional morphologic criteria with p16, p21, PR, bcl-2, p53 and Ki-67 expression may be of great value in the assessment of uterine smooth muscle tumors of uncertain or borderline histology.

## Consent

Written informed consent was obtained from the patient for publication of this Case report and any accompanying images. A copy of the written consent is available for review by the Editor-in-Chief of this Journal.

## Abbreviations

ALM: Atypical leiomyoma
bcl-2: B-cell lymphoma 2 (regulator proteins that regulate cell death; apoptosis)
CDK4: Cyclin-dependent kinase 4
ER: Estrogen
HPF: High power field
LM: Leiomyoma
LMS: Leiomyosarcoma
p16: Cyclin-dependent kinase inhibitor 2A, multiple tumor suppressor 1
p53: Tumor suppressor gene p53
PR: Progesterone
SMA: Smooth muscle actin
STUMP: Smooth muscle tumor of uncertain malignant potential
